# Next-Generation Materials for Energy Storage and Conversion

**DOI:** 10.3390/ma14030696

**Published:** 2021-02-02

**Authors:** Il Tae Kim

**Affiliations:** Department of Chemical and Biological Engineering, Gachon University, Seongnam-si, Gyeonggi-do 13120, Korea; itkim@gachon.ac.kr; Tel.: +82-31-750-8835; Fax: +82-31-750-5363

A significant amount of energy is utilized daily around the world. As a result, much research has been performed to determine highly efficient methods of storing and converting essential energy [1]. Examples of energy-storage systems that have been extensively explored for power sources with high energy/power density, a long operation lifetime, and high system stability include lithium-ion batteries, sodium-ion batteries, hybrid supercapacitors, multivalent-ion batteries, metal–sulfur/air batteries, and energy conversion systems, including the proton exchange membrane fuel cell, solid oxide fuel cell, and alkaline fuel cell [2,3]. Accordingly, a variety of device components, including anodes, cathodes, membranes, electrolytes, and catalysts, have been investigated for the purpose of improving energy storage and conversion systems, from which material design and performance optimization can be carried out.

Comprehensive research into energy storage and conversion requires a multidisciplinary approach due to its intrinsic potential to implement high-performance electrochemical systems for the real energy industry. In addition to proposing novel materials for high-performance energy systems, the stabilization of the energy systems under high-temperature conditions, as well as the optimization of the energy conversion systems, are essential. Therefore, inspiring energy storage/conversion-related research is essential for designing advanced materials and building process–structure–property relationships.

This Special Issue consists of five original, full-length articles on advanced materials for energy storage and conversion, where innovative designs for electrode materials and thermal energy storage systems, and effective experimental rationales in temperature and reactant humidification for constructing outstanding anion exchange membrane fuel cells, were introduced. M. Tangstad et al. [4] reported on the optimum content of Si-B alloy and graphite as a potential phase change material (PCM) for high-temperature thermal-energy storage systems. By investigating several important features, including the formation of carbide layers and carbon solubility when varying temperature and B content, the Si-3.25B eutectic alloy was proposed as the potential PCM in the graphite crucible. 

The performance of an anion-exchange membrane fuel cell (AEMFC) was optimized by H. Yang [5] by adjusting the operating temperature and humidifying the inlet gas. A high-performance AEMFC could be realized when elevating the cell operating temperature along with the optimization of the inlet gases at the anode and cathode. It suggested sophisticated water management for achieving high–performance AEMFC systems. Based on its outstanding findings, this article has been recorded as a feature paper in this Special Issue.

Meanwhile, three articles related to Li-ions storage have been published, providing a roadmap for next-generation energy storage. First, A. P. Nowak et al. [6] introduced a biomass, laboratory-cultivated diatom algae (*Pseudostaurosira trainorii*) for generating a 3D-structured biosilica and organic matter (the source of carbon) that can be utilized in lithium-ion battery anodes. When varying the pyrolysis temperature from 600 to 1000 °C, the structural and electrochemical properties were differentiated. It was found that the biomass pyrolized at 600 °C demonstrated the best electrochemical performance, corresponding to a specific discharge capacity of 460 mAh g^−1^ for 70 cycles. Therefore, this article paved the way for bio-silica applications as anode materials in energy storage systems. Secondly, the low-cost, lightweight, flexible, and environmentally favorable carbon nanocrystalline (CNC) was applied by S. J. Park et al. [7] as the conductive matrix for preventing aggregation of tin dioxide (SnO_2_) nanofibers in the composites. The process of heat-treatment (~800 °C) of the composite rendered it reasonable electrochemical properties, showing an initial discharge capacity of 1752 mA h g ^−1^, and it maintained a discharge capacity of ~270 mA h g^−1^ after 500 cycles. Finally, I. T. Kim et al. [8] developed a novel alloy system based on the germanium and tellurium for lithium-ion battery anode. The developed GeTe-TiC-C electrodes synthesized by a simple high-energy mechanical milling (HEMM) displayed a notable electrochemical performance compared to GeTe electrode, which could result from the formation of a conductive TiC-C matrix acting as a buffering agent against large volume changes upon cycling. The high performance of the electrodes means that they could be considered as a potential alternative to graphite for the advanced lithium-ion storage.

All the published articles were thoroughly refereed through the standard single-blind peer-review process of the *Materials* journal. As Guest Editor, I would like to acknowledge all of the authors for their excellent contributions and the reviewers for their valuable and concrete comments, which greatly improved the quality of the papers. Finally, I would like to thank the staff members of *Materials*, in particular Ms. Ariel Zhou, Section Managing Editor, for her devoted efforts and kind assistance.
